# Book Reviews

**Published:** 1810-07

**Authors:** 


### CRITICAL ANALYSIS OF THE RECENT PUBLICATIONS IN THE DIFFERENT BRANCHES OF PHYSIC, SURGERY, AND MEDICAL PHILOSOPHY.

---

*Thomæ Simsoni, Medicinæ Professoris Candossensis, in Academia Andreana, apud Scotos, de re Medica, Dissertationes quatuor. In usum Medicinæ et Humanitatis Studiosorum iterum excudi curabat Andreas Duncan, Senior, M. D. & P. Principis Scotiæ Medicus Primarius. His adnectuntur, de Asthmate Infantum Spasmodico, Dissertatio, auctore Jacobo Simson. De Alvi Purgantium Natura et Usu Dissertatio; et de laudibus Gulielmi Harvei, Oratio, auctore Andrea Duncan.* Edinburgi, 1809. 8vo. pp. 327.

Four Dissertations on Medical Subjects by THOMAS SIMSON, Chandos Professor of Medicine in the University of St. Andrew's. Reprinted by Dr. DUNCAN, for the Use of the Students at Edinburgh; to which are subjoined, a Dissertation on the Croup, by JAMES SIMSON; a Dissertation on the Nature and Use of Purgatives, and a Harveian Oration by A. DUNCAN.

PROFESSOR SIMSON, in his Preface, observes, that notwithstanding all the experience to be derived from books, all the great discoveries and improvements in anatomy, chemistry, and materia medica; enough still remains unknown in the practice of medicine, for the exercise of every one's industry and ingenuity. Many defects require to be supplied, many erroneous notions remain to be corrected, and no small quantity of rubbish to be cleared away. "I propose to myself, says he, first to inquire, with some care, what kind of remedies are commonly employed in the cure of certain diseases, and for what reasons: then, by way of specimen, to subjoin an examination of that disease, than which, none afflicts the inhabitants of this kingdom more frequently or more seriously, and which, from its exciting cause, has obtained the name of *cold.* This disease, if I am not mistaken, I have shewn not to arise from any corrupt or morbid state of the *fluids*, as is commonly believed; and at the same time, that the cure of it is not to be sought for in *incidents*, as some say, or in *dissolvents* or *deobstruents*, or any such specifics, as others believe; and that the employment of such remedies, in other diseases, has no foundation in reason, though the practice is so general." As the classing of medicines under general heads, like all other classification, is principally intended to abridge the language or the acquisition of science; and as these classes were probably at first formed to suit the different medical theories theories which happened to prevail at the time, so when the theories fell into contempt or neglect, the classes built upon them naturally participated in their fate. The language, however, of valuable original authors on the practice of medicine, would remain unchanged in their works, and consequently the terms founded upon the prevailing theory at the time they wrote. Thus in every fluctuating science, as medicine or chemistry, many terms will continue in a certain degree of use, long after the public in general have ceased to attempt a defence of their propriety. Such appear to be the terms objected to by Professor S.

The first of these Dissertations is entitled, " An inaugural discourse on the errors of both the ancients and the moderns respecting the *materia medica*." He sets out with laying down his two principal propositions or axioms. 1. " A practitioner, *as such*, cares for nothing but the effect of a medicine upon the living human body in various states of disease." 2. " An accurate possession of this knowledge is very difficult to be obtained."

By the first he means, that the origin of the metalic or vegetable substance, its natural history, its colour or chemical properties, its manipulation or preparation, are nothing to him as a *clinical* practitioner.

By the second, he wishes to impress upon his hearers, the great difficulty of acquiring a correct knowledge of the powers of medicines administered internally; and the general fallacy of medical logic.

He next proceeds to establish a third dogma, for, perhaps, this will not be received as an axiom by many of our readers; " that nothing ought to be admitted as a medical experiment, the whole and singular of the conditions of which are not clearly laid down; so that when occasion may require it, any apothecaries' apprentice might repeat it with the same confidence as a natural philosopher, properly educated, can repeat any one of Sir Isaac Newton's experiments on light and colours. For, (he adds) medical practice ought to be put out of the reach of conjecture; nor, unless in desperate cases, ought any *anceps remedium* ever to be tried."

This extraordinary opinion, if universally adopted, we think would put an end to all improvement in the practice of physic; for every *new* remedy is necessarily an *anceps* remedium. A little farther on, (§ 12,) he appears to us to invalidate his own dogma, for he says, very properly in our opinion, " All the world will readily confess, that the first origin of *any materia medica*, as well as that of the whole science of medicine, is enveloped in the greatest obscurity;" for the human mind could never have had any clue to guide it into a comparison of disease with natural productions, as the means of counteracting its fatal tendency. Hence it must follow, that the first hint of the utility of any new remedy must have been derived from accident, or from the analogy of other similar means of cure: in either case, the new remedy must long have continued an anceps remedium.

Our author, in § 16, and the following ones, enlarges on the vague notions entertained even by Hiprocates, respecting the virtues of medicines; and then downwards through Aretæus, Galen, the Arabian physicians, Holerius, Duretus, Jacotius, Plater, Riverius, Etmuller, and Sir John Floyer, who says we may distinguish the virtues of medicines by our senses.

On the discovery of the circulation of the blood, the mathematical physicians conceived that the materia medica might and ought to be founded on mechanical principles. Momentum, fluidity, density, cohesion, &c. became favourite objects of research and calculation. Friend mixed blood drawn from a vein with various substances, in order to ascertain the effect of such substances upon the blood in the human body. Professor S. exposes the futility of such experiments by arguments which no one at this time of day will be disposed to controvert. After this general sketch of the history of the materia medica and the sources from which it has flowed, he proceeds to examine it as it now stands in our systematic writers, who have treated expressly on the subject. He blames them all for omitting to give the means they used, to prove that the medicines do really possess the virtues implied by the title of the class under which they are arranged. We do not see the justice of this censure, for a large portion of all human knowledge, which does not admit of demonstration, must be founded on the mere authority of others.

As a specimen of his manner of treating these obsolete classes, we will translate his remarks on *cephalics*. "It is manifest, from the titles of the principal heads or classes under which medicines are commonly reduced, as cephalics, hysterics, hepatics, &c. without any history of the disease in which they are to be employed, that such an arrangement can answer no practical purpose. For who, from these naked titles, can determine the virtues of any medicine? In our examination of these classes, we shall readily grant what they assume, who have laboured most diligently in explaining them, and admit that by cephalics is merely to be understood, those remedies which directly refer to the brain, and which relieve disorders of the animal spirits. Let this then be the sense in which the term cephalic remedy, no matter which of them, is used; but to which, or what sort of disease of the head, is any one who reads it, to suppose it ought to be applied? Will he have no difficulty or hesitation among so many diseases of the brain, such as lethargy, apoplexy, catalepsy, paralysis, epilepsy, tremor, phrenitis, mania, and many others comprehended under the same title? Let him then, if he pleases, go to the nosologists, and learn how many different species there are of each genus, depending on different causes: and how often, almost the same affections are produced by *compression* only; whether that compression depends on inflammation, or extravasated fluid, or *plethora*, unaccompanied by obstruction: and he will easily discover how absurd it must be, in cases so various, to rely on any one *catholicon*. But there are besides, besides, many other affections of the brain, arising from poison, for instance, or opiates, (the nature of whose operation we know very little about,) in relieving which, these famous cephalics would not be worth a straw; while a draught of warm water given in time is often of the greatest utility, if repeated vomiting is produced, (as Wepfer abundantly proves in his elaborate work on water hemlock,) although the warm water does not contain a grain of any aromatic, either of odour or flavour, which all our common cephalics abound in. I do not deny that the cephalics already mentioned, may often in the more simple cases, recruit the languid powers of the body; but a prudent practitioner will not draw an argument from the effects of medicines in simple and trivial cases, to more complicated and serious ones."

But to these epithets of medicaments just related, they add others of a different kind, which because they possess more manifest and certain effects, and are placed under more appropriate titles, you may say ought to be retained: such are calefacients, soporifics, dissolvents, incrassants, purgatives, diuretics, &c. since there are diseases in which calefacients are necessary, others where soporifics, dissolvents, purgatives, &c. are of great benefit. But although these are liable to fewer objections, yet there is no small degree of uncertainty attached to them, since it is obvious that medicines of very different powers are comprehended under the same title, and that some of them are better suited to particular states of disease than others; so that the right use of any of them must depend entirely on an accurate knowledge of the history of the disease."

Near the end of this dissertation, he makes some very proper observations on the absurdity of jumbling a great number of simple remedies together in the same prescription, and thinks, Quo simplicius eo melius. This ground, however, has been so often gone over before, that nothing new can be expected.

The discourse concludes with a panegyric on their noble patron the Duke of Chandos.

*The second Dissertation* is on the natural method of curing diseases employed by the ancients before the discovery of the circulation of the blood by Harvey. Having in his first dissertation demonstrated, what he considers as the miserable state in which the materia medica was in his time; in this second, he proposes a plan for improving it. "I shall endeavour, says he, to demonstrate that great improvement may be made in the cure of diseases, if we begin with a correct knowledge of the use of remedies: but that this knowledge can only proceed from a correct and accurate knowledge of the history of diseases; for from a complete history of a disease there naturally arise certain indications of cure, by means of which we are led to remedies suitable for that purpose." "I am, continues he, the more confident in this opinion, because I observe the great founder of our science to entertain the same sentiments; a physician, he says, is sufficiently prepared to cure it who who knows the disease sufficiently: and Sydenham also, the great modern cultivator of simplicity in practice, says, 'I have often thought that if I had a clear knowledge of the history of any disease, I could always find a suitable remedy.' But not designing to accumulate great authorities only, I shall endeavour to give such an explanation of this simple plan of practice, that any one may be able to judge of it for himself." He then states the first steps which were taken to bring medicine into the form of a science, and these were merely observing and noting down the various symptoms that occurred in different stages of the same disease, and in persons of different ages or constitutions. This practice continued long before any attempt was made to adopt specific remedies, either to symptoms or diseases, unless, perhaps, cold water for thirst or febrile heat. He thinks this diligent and patient observation of symptoms, without interfering with Nature's operations, was the parent of those general conclusions respecting the event of certain signs and symptoms, called aphorisms. Before the discovery of Harvey, he thinks it would have been impossible to frame any rational theory for the practice of medicine, and that therefore the best rule was to rely on the simple palliatives or remedies which accident had discovered; that a knowledge of the history and obvious causes of any disease, together with the state of the functions at the time, were always sufficient to enable the physician to regulate the diet of the patient, which long continued to be the principal materia medica. Nature would very early point out several purgatives, astringents, sternutatories, and emetics, both in the vegetable and mineral kingdom; which, with baths, fomentations, and frictions, in conjunction with diet, would form no inconsiderable *apparatus medicaminum.*

Furnished with these instruments, and ever intent on the progress of symptoms in each disease, such aphorisms as the following would soon suggest themselves to a discerning practitioner.

1. An attack of sneezing cures the hiccup.
2. The dropsy is cured by a determination of the water to the bowels or kidnies, so is leucophlegmasia.
3. A troublesome diarrhœa is cured by a spontaneous vomiting.

Many such would be collected and treasured up to form a practical system of medicine; and such really was the system of the ancients, before anatomy or chemistry could have contributed much towards its perfection.

After this view of the simple system of the ancients, he proceeds to establish his second proposition, viz. "Diseases cannot be rationally cured by any medicines, or any auxiliary means which are not made subservient to certain indications, and directed to the fulfilment of them; any other attempt is only rash empiricism."

This proposition, which probably few will be disposed to controvert, in his sense of it, he supports by arguments of his own, and the authorities of Celsus, Pitcairn, Boyle, and Sydenham. There are, however, some diseases in which the remedies already known are are of no avail, such as hydrophobia; and surely no one would contend that we ought to remain satisfied, without attempting to discover others that are more efficacious; or else some more successful manner of employing those we have; and this we think can only be done by trials, or empiricism directed by analogy. We cannot, therefore, agree with him in the opinion that medicine may, and ought always to be cultivated precisely in the same manner as astronomy, that is, by means of observations alone. He says that Sydenham always conformed to this rule, and by so doing, advanced far beyond all his predecessors and contemporaries.

He concludes, that all rational practice ought to arise from indications, presented by an attentive study of the history and symptoms of the disease, and never to be directed by any supposed theory concerning its nature or causes. That theory, usurping the place of observation, gave birth to those absurd classes of remedies, such as acids, alcalines, solvents, incrassants, &c.

*The third Dissertation* is entitled an inquiry into the importance of the humours of the body, in a medical view, and how far they may be deteriorated by cold.

After an introduction of considerable length, in which he states the impossibility of explaining the secretions of the different fluids of the body, on the principles of chemistry, hydraulics, or hydrostatics; he lays it down as a medical fact, founded on observation, and which he denominates very properly, as we think, "*res observatu dignissima.*" "That no defective action *ever* takes place in the *solids* of the body, which is not attended with a corresponding deterioration of the circulating and secreted fluids; and that as soon as the due action of the solids is restored, the defects in the fluids begin to disappear."

Hence, he infers, we may understand why the healthy and vigorous action of the solids in the hardy rustic, produce from meagre cheese, salted and dried beef or bacon, better juices than the pampered Peer can from *living* milk and animal food, that has scarcely lost the vital principle. Having established this dependence of the sanity of the fluids on the due action of the solids, he proceeds to inquire into the effects of cold upon the condition of the humours of the body; respecting which, so many difficulties and disputes have so long agitated the professors of medicine.

Sanctorius appeared to have solved the problem by his important discovery and demonstration of the insensible perspiration, which being much impeded, or totally obstructed by cold, must deteriorate the humours, by retaining effete or pernicious portions of them within the body, which ought to be carried off by that outlet.

This highly popular theory was first assailed by our countryman Dr. Keil, who contended on the other hand, "That some noxious principles were introduced into the system by the exposure to cold, which coagulated the fluids, and produced those aggravated symptoms which Sanctorius ascribed to the retention of excrementitious fluids.

Our author decidedly prefers the theory of Dr. Keil, which, he says, corresponds with the ancients, and blames Sanctorius for rejecting it. He, however, rejects both, and proposes the following one of his own. "Nature has established so just and nice a balance between the circulating powers of the heart and arteries, and that of the returning veins and secreting organs, that no impediment can take place in any of the latter, without great and manifest derangement in the former." For the demonstration of this fundamental proposition, he refers to Theophilus Benedictus, and the injections of Ruysch, which, he says, sufficiently prove, "that any extraordinary power pressing upon, or urging the extremities of the circulating vessels, must necessarily produce great *extravasations* of fluids in every part of the body." Here we see that this enemy of theoretical speculations, is indulging in one as wild, and apparently as ill-founded, as any of those that he so severely condemns.

Taking his theory for granted, and the well-known properties of cold in condensing all fluids, especially the animal fluids; he says, all the symptoms arising from cold, will admit of a very easy explanation. For the most remarkable effects of cold, are catarrhs and glandular tumours, in which the secreting vessels are impeded in their action by the debilitating powers of this pernicious agent, while the blood retiring from the surface, is thrown with greater force on the internal parts. The illustration and confirmation of this theory is as wild as the theory itself. The whole rests upon lentor, elasticity, and those applications of mechanics, hydraulics and hydrostatics, which became so fashionable for some time after the discovery of the circulation. Defluxions from the head and bronchiæ are made to depend much more on the moisture combined with the cold, than on the cold itself, and appeals to our feelings in confirmation of the opinion.

His plan of cure in diseases proceeding from cold, is precisely the same as that of all rational practitioners, whatever their theory may have been, or in whatever times they may have lived. He employs blood-letting, general or topical; laxatives, low and diluent diet; and the most effectual means of restoring a free circulation in the obstructed vessels on or near the surface, by warmth, friction, fomentations, blisters, &c. He does not omit, however, to guard us against the danger of pushing any of these so far as to induce inordinate debility in the patient.

After these means have been employed, if a rheumatism should assume the chronic character, he advises chalybeates and other *tonics*. In local inflammations proceeding from cold, he thinks local remedies will be sufficient.

*The fourth Dissertation*, is a continuation of the preceding. In this the professor combats the humoral pathology, which makes diseases to depend upon, and arise from, a depraved state of the fluids, instead of the imperfect action of the solids.

The opinion of the professor is now so universally received, that we we do not think it necessary to do more than point out the foundations of his several arguments in support of the muscular and nervous pathology, in opposition to the humoral. This subject, indeed, has been so ably discussed by Hoffman, Cullen, Brown, Beddoes, and Darwin, that we need only to refer to their works. He grounds his arguments, in the first place, on *sympathy*, which cannot be supposed to depend on the fluids. The instances of sympathy, which he principally lays a stress on, are those arising from an irregular or defective action of the uterus, in chlorosis, and emansio mensium; increased action of the uterus and its vessels in pregnancy and menorrhagia, which are known to affect the functions of the stomach, the action of the heart, and the secreting glands of the whole body.

Hysteria is with reason much dwelt on, as this can have nothing to do with the humoral pathology, and must be referred to the mind or sympathy.

The stomach and heart are liable to be affected by passions of the mind so violently, as to have their actions suspended or inverted; and the former is often sympathetically affected by the diseases of the kidnies, ureters, bladder, or urethra. Spasmodic diseases, as may be expected, are not overlooked, of which no one suspects any derangement of the fluids to be the cause.

The liver and its secretion are laid much stress on by the supporters of the humoral pathology; Dr. Simpson, therefore, enters into this subject at considerable length, and explains the derangements supposed to depend on the vitiated state of the bile, to arise from its quantity, or the obstructions to its excretion; that diarrhœa, or the opposite state of the bowels, depends on muscular and nervous action, not on the quality of the fluids; and concludes his dissertation, by referring the effects of emetics, purgatives, and poisons, to their stimulant action on the solids, not to any change they can possibly produce in the fluids in their primary operation.

*The Thesis on Croup*, by Dr. James Simpson, printed in 1761, contains nothing interesting to practitioners at present; and the same may be said of the Thesis on the nature and use of purgatives.

Such are the contents of these Latin Essays, written long ago, which Dr. Duncan has judged it proper to re-publish in 1809. Whether they are re-published on account of the matter they contain, or as a model of style for the imitation of the students, for whose use it is now printed, we leave to those who may read them to determine.

*A Practical Essay on Cancer; being the Substance of Observations to which the Annual Prize for* 1808 *was adjudged by the Royal College of Surgeons of London.* By CHRISTOPHER TURNER JOHNSON, Surgeon, Exeter; Member of the Royal College of Surgeons of London, and of the Royal Medical Society of Edinburgh. 8vo. pp. 126. London, 1810.

THAT Cancer has long been esteemed one of the opprobria medicorum must be admitted, and every attempt to investigate its history and to improve its treatment, deserves encouragement; it is upon these grounds, therefore, we presume, the Royal College of Surgeons have bestowed their annual medal for this performance. How far the author has been successful in effecting either one or the other of these objects, or whether he has, in the least degree, added to our previous knowledge of the subject, it is our duty to ascertain, taking it for granted that nothing superior to the present Essay was offered to the notice of the Royal College on this occasion.

"The term Cancer," the author informs us, in the first paragraph of his book, is employed "to denote a particular connected train or series of morbid phenomena, occurring in the human body." We can inform the author, that the terms gout, small-pox, fever, and the name of every disease incident to the human body, are so employed; but surely it becomes necessary to enumerate, at least, *some* of the phenomena occurring in this particular train, to constitute the definition of any one disease; and yet, that the author means this paragraph to stand for a definition, we conclude, because we can find *no other*. In appropriating terms, indeed to the various stages of the disease (whatever it be) he is treating of, he thinks it requisite to ascertain the extent of signification in which they should be applied to the different stages respectively, and which he does as follow:

"The words schirrhus, carcinoma, and cancer, have been variously employed, in extent of signification, by almost every different writer; and from this circumstance, it becomes necessary to explain the meaning which is intended to be attached to them in the subsequent parts of this Essay. Cancer, as a general term, will be used to express every gradation in the disease, from its commencement to its termination; but, as it may sometimes be desirable to speak in a more limited sense, I shall, for this purpose, and in compliance with custom, distinguish two principal stages of the disease;—the *scirrhous* and *carcinomatous*."

Here the word cancer being used as a general term to express every gradation in *the disease*, remains equally undefined as to what particular train of phenomena it is applied, and we are equally in the dark as to the precise disease the author means to treat of. We were the more surprised at this want of precision in the author, because he observes, "Under the title of cancer, it has been but too frequent a practice, to associate together an almost indefinite number of morbid alterations of structure, without previously viously inquiring whether they possessed any such settled agreement of character, as would, with strict propriety, allow of individual cases of disease being included under a general definition. This has most unquestionably been one very fertile source of error; and, while speaking of it, I may, therefore, advert to the necessity which there exists in every philosophical investigation, of setting out with a clear and distinct conception of the particular object of research; for, in what case, let i. be asked, ought this mode of reasoning to be more forcibly insisted on, than where our enquiries relate to the nature and cure of disease? These, it deserves well to be recollected, are matters which involve considerations of much more than speculative importance."

To conduct a philosophical investigation to any useful purpose, requires a closeness of reasoning, and an accuracy of expression which our author has not displayed in this Essay; and the obstacles which, in the following quotation, he laments as impeding the progress of our knowledge, he has by no means contributed to remove

"The promiscuous and *unguarded* use of the term cancer, has tended more, perhaps, than any other single cause to retard the progress of our knowledge concerning those affections which really deserve the appellation. No one ever pretended that we should be warranted in applying the same epithet, indiscriminately, to every different case of disease, merely on account of its proving untractable: and yet, with respect to the subject of the present inquiry, this very thing seems to have been done, without the least scruple or hesitation. It is sufficiently obvious, however, that in order to constitute the identity of any two cases of disease, something more must be required than such vague and very distant features of resemblance."

Were we inclined to cavil, we might observe, that the circumstance of a disease proving generally untractable, is sufficient to justify us in bestowing, "without the least scruple or hesitation," a pretty harsh *epithet* upon it; but no epithet can amount to a definition of a disease, it does not even constitute its name, being never more than an adjunct to distinguish some good or bad quality belonging to it; we agree with the author that no features of *resemblance* can constitute the identity of any *two* cases.

"The author's aim, is to collect and concentrate into one point of view, all the valuable information that is to be found scattered throughout the works of preceding writers on Cancer;" but he informs us, that "there are yet, comparatively, but few cases of cancer upon record, which have not lost more or less of their sterling value, by want of due attention to some of the particulars which have been here mentioned." To whom this want of due attention, whereby these cases have *lost* their sterling value, is to be attributed, we do not discern; we ourselves should rather have judged these cases to have been *originally deficient* in sterling value, from this cause; but we submit to our author, who thinks otherwise, wise, for that he has correctly expressed himself we cannot doubt, since in the very next paragraph he says, "it is by giving precision to our language, and *by this alone*, that we can ever hope to obviate those frivolous and unmeaning disputes, which turning upon the different interpretation of *particular words* and phrases, infest medical science, in common with almost every other branch of useful knowledge." We have before observed, how desirable it is, that in every case, correct definitions should be given of the diseases on which authors mean to treat; we presume Mr. J. means to express the same opinion in the following paragraph, although, we confess, we do not clearly understand what is meant by establishing a *more intimate connexion* between the *word* cancer, and the particular phenomena of which it *should be* expressive.

"An attempt is still worth making to establish a more intimate connexion than has hitherto subsisted, between the word cancer and the particular phenomena of which it should be expressive. This would require that its precise extent of signification should be clearly and accurately defined: which, though it might have the effect of excluding from the catalogue many diseases vulgarly called cancerous, must yet be a principal means of promoting careful and accurate observation. In prosecution of this plan, it is likely that we should in time become better qualified to mark the nicer distinctions, and shades of difference, which separate this complaint from every other."

We have dwelt thus long upon this part of our author's book, because we considered it our duty, particularly, to examine a publication ushered into the world under the auspices of the Royal College, in order to ascertain what claims the author had upon the attention of the public, independent of collegiate sanction. The intrinsic merits of the work do not consist in a superior philosophical arrangement of the various parts of the subject, nor in a more luminous view of the facts already known; but as the result even of individual experience is always valuable, when related in language correct enough to be easily understood, we shall proceed to detail the substance of our author's information on the subject before us.

The chapter on the History of Cancer is divided into several sections, detailing, respectively, the progress of the symptoms in the various parts of the body which may be affected with the disease, as the breast, the uterus, the testicle, &c. In the term Cancer, the author includes two stages, the schirrhus and carcinomatous; scirrhus, however, may subsist independent of any cancerous affection, either local or constitutional, nor do we think that the author has used the term cancer in the general acceptation, since all nosologists have arranged scirrhus and cancer as two separate affections, the former of which may exist in glandular parts as an effect of simple inflammation; but admitting that scirrhus may also be the consequence of cancerous affection, how is it to be distinguished from what may be called simple scirrhus, or what what the author calls "the enlargement of the glands from simple irritation." Speaking of *cancer* of the testicle, the Author says, "the disease *begins* here as in all other glandular organs, by a partial induration." Now as partial induration may, we conceive, arise in an organ independent of cancerous affection, how shall we distinguish when it is, or is not, cancer? Here, we think, the author has failed in giving us any diagnostic signs whatever; the same thing may be said of "that enlargement which is so frequently seen to arise in scrofulous constitutions."

"In every case of this kind, in which there may be a doubt, it will at any rate be proper to examine the state of the lymphatic glands in other parts of the body; and more particularly in the neck and axilla of the opposite side. The information which may, in this way, be obtained, taken in combination with the general history and appearance of the patient, will materially assist us in ascertaining the nature of the case. The peculiar kind of shooting pain, which is generally attendant on scirrhous structure, may also serve to facilitate the distinction.

"It has been said, that such of the lymphatic glands as are situated in the immediate vicinity of the diseased part, may undergo a degree of enlaregment from simple irritation. This, however, never seems to happen except where some accidental inflammation is present in the early stages of a scirrhus: besides which, it further admits of being distinguished, by yielding to those means which are calculated to lessen and subdue inflammation in ordinary cases."

To found any diagnosis upon the *effect* of treatment is unphilosophical, to denominate that cancer which is untractable, and to infer a similar appearance not to be so, because it gives way to certain means, is not sound reasoning; and yet, that the author depends for his diagnosis rather upon the *event*, than the actual state of such affections appears probable, when he says, "External disease, appearing under the form of either tumour or sore, will sometimes, from accidental causes, assume an appearance of malignancy which it does not in reality possess: and, viewed in this state, there is reason to believe that various other morbid affections have at different times been mistaken for cancer, and treated accordingly."

How we are to distinguish the actual degree of malignancy but by the external appearances, we are at a loss to tell; the author says, "in proportion to the rapidity of the progress of any individual case, so is its degree of malignancy." This we conceive to be a fallacious criterion, for accidental circumstances may accelerate or retard the progress of cancer, without at all affecting its malignancy, so far as is distinguishable by any symptom; and to assume rapidity of progress as a criterion, is, at least, begging the question. The history of cancer of the breast, of the uterus, and of the testicle, affords nothing very new, as the progress of the disease in each of these parts is well known.

The following description of Cutaneous Cancer, and the marks of distinction between it and Lupus, as described by Dr. Willan, deserve attention.

" Cutaneous cancer, at its commencement, usually appears under the form of a small præternatural enlargement, or elevation of the skin. In feeling and consistence it is sometimes so hard as to approach to the nature of horn; while, on other occasions, it will bear a much nearer resemblance to a common wart. There are, besides these, some few instances, in which it would look to be no more than a little discoloured pimple.

" Whatever form the disease may at first assume, there will invariably be found to take place, a degree of surrounding hardness seldom or ever to be met with, under other circumstances. Some degree of shooting pain is likewise, from time to time, experienced in the part. In many of these cases, ulceration seems to be materially accelerated by the accidental irritation of the patient's fingers, which are very often, though unconsciously, employed in the vicinity of the disease. Sometimes, however, a sort of scale is generated, so as to form a compleat covering of defence to the little tumour, and this will be artificially removed, and again renewed several times in succession, before ulceration is fairly established. This phænomenon is seen most remarkably in that affection of the scrotum peculiar to chimney sweepers.

" When the part has once arrived at a state of ulceration, it soon puts on those characters of malignancy, which have occasioned it to be classed as a species of cancer. The surface of the sore possesses, indeed, the common appearances of carcinomatous ulceration: and there is also a discharge from it, of sanious, or otherwise ill-conditioned, matter."

" The particular situations which cutaneous cancer is most frequently observed to occupy, are the lower lip, the angles of the eyes, and the alæ nasi; but some of these parts are also liable to another affection, which, though, in its nature, very widely remote from cancer, still bears a good deal of resemblance to it, in appearance. What I here allude to is a disease which has been described by Dr. Willan, under the generic name of Lupus. It begins under the form of numerous small brown pustules, which soon go on to a state of ulceration. The parts upon which it commonly first seizes, are the forehead, the eyebrows, alæ nasi, and upper lip. When this disease is situated in the nose, it is not the soft parts alone that suffer from its destructive effects; these will, in the course of time, be extended so as to affect both the cartilages and turbinated or spongy bones.

" The ulcer of the soft parts extends slowly, having a dusky, copper-coloured disk, and attended likewise by a good deal of surrounding hardness.

" This disease may generally be distinguished from cancer by a careful attention to the following circumstances. Lupus is chiefly seen to occur in young people; while, on the contrary, the cutaneous cancer is scarcely ever observed, except in those who are considerably advanced in life. Cancer, again, hardly ever attacks the upper lip, though it is perhaps one of the most frequent seats of of lupus. The pain too, in cases of lupus, is never very violent, or acute; and indeed the disease itself generally admits of being cured in no very great length of time, by means of mild external applications."

"The organ of vision is another important part, which is subject to a disease that has received the appellation of cancer." This implies a doubt in the author's mind, whether the disease, the symptoms of which are very briefly described, is really cancer; it differs, he says, from almost every other cancerous affection, in being generally a disease of early life. Several writers have noticed this disease, and Mr. Wardrop has lately given us some excellent observations upon it, under the name of Fungus hæmatodes; it is also sometimes called soft cancer. It certainly differs essentially from cancer, and should not have been included under that title.

After a chapter on scirrhous structure, in which is nothing very important or new, as it contains little more than Mr. Home's description of the appearances on dissection of cancerous scirrhus, we come to the author's "Theory of Cancer, on which we shall offer a few remarks.

The author declines entering into a formal discussion, respecting the nature and causes of cancer; the principal inquiry to which he wishes to restrict himself is, "how far is cancer, at any time, or under any circumstances, to be regarded as a local or as a constitutional disease? As far as we can understand him, cancer may be considered as a local disease, arising from constitutional predisposition; this predisposition he illustrates by the analogy of two well-known diseases, gout and scrofula; "these may, without any great impropriety of language, be called constitutional diseases; and yet, for the most part, there is reason to consider their effects as being at first of a local nature."

On the subject of predisposition, we shall quote the author's own words: "Predisposition to disease may be of two kinds; either hereditary, or acquired. In using the term acquired predisposition, I only mean to express a simple matter of fact, viz. that what are called hereditary diseases may suddenly appear in a family where, after the most careful research, no traces whatever of their previous existence can be discovered. Now, in this case, we must either allow that a predisposition is first acquired, or that the accidental causes have been applied so powerfully as to excite the disease in a constitution otherwise sound. Supposing, however, that the disease be at first excited in this casual manner; yet the person who is the subject of it, will afterwards be as fully capable of transmitting the predisposition to his descendants, as one who shall have inherited it from a long train of ancestors."

This appears to us very questionable; that a strictly local disease, produced by accidental external violence, as by a blow on the breast, in "a constitution otherwise sound," should confer a power on the individual to transmit to his descendants an hereditary tary predisposition to the same disease, is an hypothesis neither supported by facts, nor warranted by analogy; it is also contradictory to what the author had before advanced in his attempt to prove, that cancer is not a mere local disease, from its sometimes attacking different parts of the body at the same time.

"It cannot be urged, in opposition to this, that such parts may have been equally exposed to be affected by external or accidental causes; because these same causes, admitting their agency, would at most, in *other constitutions*, only have given rise to *simple inflammation, and its consequences.*"

If simple inflammation and its consequences only, can be produced from external accidental causes, in other constitutions, how can the hereditary cancerous predisposition ever be *acquired* from the most violent application of these causes?

Among the predisposing causes of cancer, the author enumerates climate, this disease being much more frequent in cold countries, and proportionally rare in warm ones; we confess, we do not understand the following note, nor comprehend the practical difference between arresting and retarding. "*Query*, Though a warm climate may not be sufficient to *arrest* the progress of the disease, under these circumstances, yet will it not *retard* it?"

The most important part of a "Practical Essay" is undoubtedly that, which explains the treatment of the disease, and to this we shall now turn our attention.

We were particularly struck with the following paragragh; as we think the comparative results of the author's experience, would be better adapted to a practical essay, than speculative reasoning upon facts already known.

"The author has purposely avoided introducing any detail of the cases which have fallen under his own particular observation, as he did not conceive that they were calculated to prove any thing more than what is met with in the ordinary course of practice; and in point of numbers could not be put in competition with the extensive scale of facts already brought forward."

The treatment of cancer must be either by internal medicines or external applications; in regard to the former, all discussion is vain, if the following observation is true; and we must, as our author has done, confine ourselves to the external treatment of the disease.

"It would be a very tedious, and at the same time an unprofitable task, to attempt to offer a detailed account of the various articles of the Materia Medica, that have been given internally with a view to the cure of cancer. Let it, therefore, suffice to observe, that there is not a single medicine of any general efficacy, but what has at some time or other been exhibited, under every variety both of form and circumstances. The collective experience of past ages, however, serves only to shew, that, so far as respects a cure, all such means are equally devoid of efficacy."

It

It is very extraordinary, however, that a disease depending so generally, according to the author, upon constitutional predisposition, should be altogether uninfluenced by internal remedies. A permanent cure then can only be effected by local applications, which will completely remove the tumour, or by excision; and the author does not hesitate to give a decided preference to the latter method; but we will state his opinion in his own words.

"I shall now then proceed briefly to notice those means of cure which have been made use of in cancerous affections, externally.

"The impractibility of reducing the absolute size of a truly scirrhous tumour, is a circumstance which has already been noticed in a former part of the Essay. All the various applications, therefore, which have been made use of to parts affected with cancer, have been intended to fulfill nearly the same indications of cure; viz. to destroy the living powers of the morbid growth, and to effect its consequent separation from the sound and living parts which lie immediately adjacent.

"The question accordingly comes to this issue, whether any of the articles which have been employed for the above purpose, possess a power capable of accomplishing such intentions?

"It would not be right to deny that, under certain circumstances, they may possess such a power; but, if we reflect seriously on the great inconvenience, danger, and uncertainty, which necessarily attend their operation, they will, it is conceived, deservedly appear to be held cheap in the general estimation of the profession.

"To place this subject in a more familiar point of view, let us, by way of illustration, suppose an application of this kind to be made to a scirrhous breast. Can there, I would ask, be any one credulous enough to suppose that the surgeon is endued with a discretionary power, which can be exercised over the action of such a substance? I have not called it by the name of caustic, lest a verbal objection should be raised against the use of the term. Is it for a moment to be supposed, that an application of this sort can be so determined in its operation, as completely to destroy the morbid parts, without fear of injury to others, where its effects might not only prove highly prejudicial, but even dangerous to life? I should, without the smallest hesitation, answer both these questions decidedly in the negative. Who is there, I would further ask, that could pretend to say, with any certainty, when the whole of a scirrhous tumour had been removed by these means?

"These, however, are not the only objections to the use of such local applications. Their operation is attended, for the most part, with excessive pain, and a high degree of inflammation; besides which, in order to ensure the very precarious prospect of success, which their use affords, it often becomes necessary to repeat them several times. I shall not at present say any thing about the difficulty of healing an extensive sore, formed in this way; because the great objection to all similar means of cure is derived from the long continued inflammation with which they are necessarily attended. This inflammation tends very considerably to hasten the further extension of the disease, through the medium of the lymphatic vessels; and so put it, in a little time, altogether beyond the reach of art.

"The only other method by which it has been attempted to effect a permanent cure, consists in the excision of the diseased parts: and, though we have but too often to lament its failure when performed late, yet, even under the most unfavourable circumstances, it would not, I conceive, be difficult to shew its advantages over every other proposed means of relief."

The excision being determined upon, a question arises, at what period of the disease should the operation be performed; the author contends for an early removal, and endeavours to refute Mr. Pearson's objections to its being indiscriminately had recourse to in the early stages of the disease. Mr. Pearson approves of early extirpation when the whole of the diseased parts can be removed, but he thinks the chance of success greater when the disease is at a stand and its extent can be ascertained, than when it is in progress, and therefore proposes that early excision should be adopted with some limitations. Here we think the author's work defective, and we wish he had informed us, from his own experience, of the comparative results of the operation performed in various periods and stages of the disease.

The author next describes the manner of performing the operation on the various parts affected with cancer; for this, we must refer our readers to the work itself; having already sufficiently extended our remarks, we shall only mention, that in *every case* of scirrhous breasts, he recommends the removal of the *whole* of the breast, whatever be the extent of the disease. This is, certainly, an effectual method of preventing that individual organ from being again affected; but as it does not remove the constitutional predisposition, hereditary or acquired, and without which predisposition, the author says, *simple inflammation only*, not cancer, could have been at first produced, the patient is not at all secured from a recurrence of the disease in some other part.

---

*A Conspectus of the Pharmacopœias of the London, Edinburgh, and Dublin Colleges of Physicians; being a Practical Compendium of Materia Medica and Pharmacy.* By A. Todd Thomson, Fellow of the Medical Society of London, and of the Medical, the Royal Physical, and the Speculative Societies of Edinburgh. 12mo. pp. 283.

This little volume has made its appearance at a time when it is likely to be acceptable to the members of the medical profession, as it contains all the new articles of the materia medica, and the officinal preparations inserted by the London College in their last Pharmacopœia, and may serve as an abridgment of the larger work work of the same kind, published by Dr. Duncan, jun. Under each article of the vegetable kingdom, the place it holds in the Systems of Linnæus and Jussieu is stated, its original place of growth pointed out, and the terms of its existence marked in the characters used by botanical writers. The chemical components of the different substances are taken from Murray's and Thompson's Systems of Chemistry; and the properties of most of the vegetable productions from the Materia Medica a Regno Vegetabili of Bergius.

With regard to the medical properties and doses, although we should advise those never to presume to prescribe remedies who have not laid a deeper foundation than can be acquired from a Conspectus or Compendium, yet, as the precise dose of a medicine, not constantly used by any one, is frequently forgotten, this little Manual affords a ready opportunity of recalling it to memory. Under each article, also, is added, what we think valuable information, the various substances with which that article ought not to be joined in composition; for we must all have seen absurd blunders sometimes committed in prescriptions even by physicians of some eminence, for want of knowing, or of recollecting, the chemical affinities of the several substances prescribed for medicines.

